# Scalp Involvement in Primary Cutaneous Lymphomas—An Update on Clinical Presentation, Diagnostics, and Management

**DOI:** 10.3390/cancers17101678

**Published:** 2025-05-16

**Authors:** Karol Kołkowski, Martyna Sławińska, Beata Zagórska, Roman J. Nowicki, Jerzy Jankau, Małgorzata Sokołowska-Wojdyło

**Affiliations:** 1 Department of Dermatology, Venereology and Allergology, University Clinical Centre, Smoluchowskiego 17 str., 80-214 Gdansk, Poland; martyna.slawinska@gumed.edu.pl (M.S.); beatazagorska@gumed.edu.pl (B.Z.); roman.nowicki@gumed.edu.pl (R.J.N.); mwojd@gumed.edu.pl (M.S.-W.); 2 Department of Plastic Surgery, University Clinical Centre, Smoluchowskiego 17 str., 80-214 Gdansk, Poland; jjankau@gumed.edu.pl; 3 Department of Dermatology, Venereology and Allergology, Medical University of Gdansk, Smoluchowskiego 17 str., 80-214 Gdansk, Poland; 4 Department of Plastic Surgery, Medical University of Gdansk, Smoluchowskiego 17 str., 80-214 Gdansk, Poland

**Keywords:** primary cutaneous lymphomas, scalp involvement, alopecia, B-cell lymphomas, T-cell lymphomas

## Abstract

Primary cutaneous lymphomas (PCLs) constitute a heterogeneous group of rare diseases. Previously, few studies have focused on the aspect of scalp involvement by PCLs. The objective of this study was to analyze the clinical presentation, diagnostic pathways, and treatment methods in patients diagnosed with scalp PCLs. A comprehensive literature review was conducted using the PubMed database, with the search terms “scalp” AND “cutaneous lymphoma”, “folliculotropic mycosis fungoides” AND “scalp”, “trichoscopy” AND “lymphoma”, and “dermoscopy” AND “scalp” AND “lymphoma.” The search was limited to articles published from database inception to May 2, 2024. Based on the title and abstract analysis, we included articles on PCLs involving the scalp. After a thorough review of the full manuscripts, several were excluded due to irrelevance, the absence of essential clinical data, discrepancies in patient age, gender, and diagnosis, and a lack of information pertinent to scalp PCLs. The literature search identified 1482 patients with scalp involvement in PCLs. Of the total number of cases, 1096 were diagnosed with B-cell PCLs, 384 with T-cell PCLs, and two cases lacked a precise PCL diagnosis. Primary cutaneous follicle center lymphoma was the most frequently reported B-cell PCL of the scalp, while mycosis fungoides was the most common T-cell PCL. Alopecia was observed in 69.0% of the patients analyzed, with the most prevalent form being non-scarring focal alopecia. It is imperative to consider the scalp in patients with PCLs, particularly in light of the knowledge that some lymphomas affecting the scalp exhibit a higher degree of aggressiveness.

## Simple Summary

Primary cutaneous lymphomas are a group of rare diseases. It is uncommon for these diseases to affect the scalp. The goal of this study was to understand how this disease looks, how it is diagnosed, and how it is treated in patients with primary cutaneous lymphoma of the scalp. A thorough review of the existing literature was performed using the PubMed database. The search terms included “scalp” and “cutaneous lymphoma”, “folliculotropic mycosis fungoides” and “scalp”, “trichoscopy” and “lymphoma”, and “dermoscopy” and “scalp” and “lymphoma.” The research found 1482 patients with skin problems caused by primary cutaneous lymphoma. Of the total number of cases, 1096 were diagnosed with B-cell primary cutaneous lymphoma, 384 with T-cell primary cutaneous lymphoma, and two cases lacked a precise diagnosis. Primary cutaneous follicle center lymphoma was the most commonly reported type of B-cell lymphoma on the scalp, while mycosis fungoides was the most common type of T-cell lymphoma. Hair loss was seen in 69.0% of the patients in this study. Some lymphomas affecting the scalp are more aggressive. Therefore, it is important to remember that primary cutaneous lymphomas may affect this area.

## 1. Introduction

Primary cutaneous lymphomas (PCLs) are a rare form of T-cell and B-cell extranodal non-Hodgkin lymphomas originating in the skin [1,2,3]. The location of PCLs may be any skin anatomical site, although in some cases, specific sites affected by PCLs have been previously associated with different prognoses. For instance, in the case of leg-type primary cutaneous diffuse large B-cell lymphoma (PCDLBCL), the prognosis is worse when the lesion is localized on the lower limbs, while in the case of primary cutaneous marginal zone lymphoma (PCMZL), the scalp has been associated with worse treatment outcomes [1,2]. Recently, indolent and aggressive variants of folliculotropic mycosis fungoides (FMF) requiring different treatments have been distinguished [4]. Some of these tumors, which have a predilection to appear on the scalp may present a different course and require modified treatment methods [5]. The epidemiological aspects, therapeutic approach, and prognostic significance of scalp skin involvement by PCLs remain poorly understood. Therefore, our aim was to provide a balanced update based on the literature on the advances in the field of PCLs affecting the scalp.

## 2. Materials and Methods

A comprehensive search of the literature using the PubMed (https://pubmed.ncbi.nlm.nih.gov/ (accessed on 2 May 2024)) electronic database using the search queries “scalp” AND “cutaneous lymphoma”, “folliculotropic mycosis fungoides” AND “scalp”, “trichoscopy” AND “lymphoma”, and “dermoscopy” AND “scalp” AND “lymphoma” was performed in the first week of May 2024, from the database inception to the 2nd of May 2024. After the initial search, titles and abstracts were screened for the inclusion and exclusion criteria. Based on the title and abstract analysis, we included articles concerning primary cutaneous lymphomas involving the scalp. At this step, we excluded records not related to the topic, non-English manuscripts, personal opinions, and duplicates. The remaining were qualified as eligible for full-text reading. Tumors arising from the head and neck area, apart from the scalp, and all secondary cutaneous lymphomas, have not been analyzed. After reading the full manuscripts, some were excluded (not relevant, lacking clinical data (age, sex, and diagnosis of a patient, and not providing information concerning primary cutaneous lymphomas of the scalp region). Additional relevant, eligible records identified through a reference search were included in which information on the primary cutaneous lymphomas of the scalp was identified. Finally, a total of 163 papers were selected for inclusion in this review, and a total of 1482 cases of patients were incorporated into the review. Data on age at the disease onset, sex, final diagnosis, alopecia in the course of the lymphoma and death related to the lymphoma, immunosuppression, skin infections, skin cancer, melanoma, medication, Borrelia burgdorferi infection, Helicobacter pylori colonization of the stomach, influenza or viral hepatitis A vaccination, arthropod bites, traumatic injuries, tattoos, gastrointestinal disorders, and autoimmune diseases in read papers have been analyzed and summarized. For the purpose of this analysis, we categorized identified cases based on the diagnosis in the following manner: mycosis fungoides (MF), Sézary Syndrome (SS), Lymphomatoid papulosis (LyP), primary cutaneous anaplastic large cell lymphoma (pcALCL), other cutaneous T-cell lymphoma (CTCL), primary cutaneous marginal zone lymphoma (PCMZL), primary cutaneous follicle center lymphoma (PCFCL), primary cutaneous diffuse large B-cell lymphoma (PCDLBCL), and other cutaneous B-cell lymphomas (CBCLs).

## 3. Results

### 3.1. Frequency and Epidemiology of Scalp Involvement in PCLs

Our literature search identified 1482 patients with reported scalp involvement in the course of PCL, 1096 of which have been diagnosed with CBCLs, 384 with CTCL, and, in 2 cases, the origin cell was unknown [1,2,4,5,6,7,8,9,10,11,12,13,14,15,16,17,18,19,20,21,22,23,24,25,26,27,28,29,30,31,32,33,34,35,36,37,38,39,40,41,42,43,44,45,46,47,48,49,50,51,52,53,54,55,56,57,58,59,60,61,62,63,64,65,66,67,68,69,70,71,72,73,74,75,76,77,78,79,80,81,82,83,84,85,86,87,88,89,90,91,92,93,94,95,96,97,98,99,100,101,102,103,104,105,106,107,108,109,110,111,112,113,114,115,116,117,118,119,120,121,122,123,124,125,126,127,128,129,130,131,132,133,134,135,136,137,138,139,140,141,142,143,144,145,146,147,148,149,150,151,152,153,154,155,156,157,158,159,160,161,162,163,164]. Primary cutaneous follicle center lymphoma was the most commonly reported primary CBCL involving the scalp, while MF was the most common primary CTCL. Among all cases analyzed, gender was reported in 502 (33.9%) patients, and significant male predominance was found. Details on the demographic data of the analyzed patients have been presented in Table 1.

The literature regarding the epidemiology of scalp involvement in CTCL is scarce. Among all studies identified by the aforementioned search strategy, only 3 original studies with more than 20 CTCL cases have been analyzed to estimate the frequency of scalp involvement in particular CTCL subtypes [4,69,134]. In the aforementioned studies, we have identified 237 cases of FMF (91.2%), 17 cases of MF (6.5%), and 6 cases of SS (2.3%). The 2018 WHO-EORTC classification underlines the tropism of FMF to the head and neck region, which is the preferential localization of these skin lesions [2].

Conversely, a United States population-based analysis has been performed regarding primary CBCL in which 4758 patients have been analyzed [1]. Both PCFCL and PCDLBCL have been found to occur significantly more often on the scalp [1]. In the case of PCFCL and PCDLBCL, 37.0% and 22.2% of lesions have been localized on the scalp based on the site of biopsy, respectively [1].

In both groups (CTCL and CBCL), male predominance was noted, with mean age at disease onset in the fifth decade of life for CTCL patients, and in the fourth decade of life for CBCL patients. The female/male ratios have been 0.6 for the CBCL group and 0.5 for the CTCL group, which is generally in line with the literature [165]. The mean age of CTCL patients appears to be similar to that reported in the literature, particularly when one considers that a significant proportion of this group is patients with FMF, who are on average diagnosed 10 years earlier than MF patients [165]. However, patients with CBCL have similar ages to those with CTCL, with the exception of patients with PCDLBCL [165]. In patients in their 70s and 80s, CBCL appeared, with the leg type being more frequent in women [165]. In our study, the mean age of disease onset was 49.1 years.

In our study, CBCL patients constitute nearly 74% of the total number of patients, which is probably a reporting bias due to two big studies on CBCLs, in which nearly 1000 patients have been reported [1,6]. PCMZL is uncommonly found in the head and neck region, while the opposite observations were made according to other subtypes of CBCLs. PCFCLs and PCDLBCLs are more likely to present on the scalp and neck [1]. Importantly, PCDLBCL is tropic to the head, neck, and scalp rather than the leg, as it was traditionally believed [1,131,132,133]. This seems to be reflected in the results of our analysis as the most frequent CBCL seems to be PCMZL, while in our study, it constitutes less than 10% of cases [163]. A comparison of CBCLs and CTCLs with scalp involvement is presented in Table 2.

### 3.2. Pathogenesis of PCLs Involving the Scalp

Despite numerous previous research, the precise pathogenetic background of PCLs remains unknown [2]. Despite checking every case for the data on age at the disease onset, sex, final diagnosis, alopecia in the course of the lymphoma and death related to the lymphoma, immunosuppression, skin infections, skin cancer, melanoma, medication, Borrelia burgdorferi infection, Helicobacter pylori colonization of the stomach, influenza or viral hepatitis A vaccination, arthropod bites, traumatic injuries, tattoos, gastrointestinal disorders, and autoimmune diseases, we did not identify any significant correlation. It remains to be elucidated as to why particular PCLs involve the scalp region more frequently.

### 3.3. Scalp Involvement as a Prognostic Factor in PCL Patients

There is a paucity of data regarding the prognostic significance of scalp involvement in the course of PCLs. Due to the fact that in the classic form of MF, the scalp is involved mostly in the advanced stages of the disease, these cases could be associated with a worse prognosis [2,5]. Alopecia was also theorized to be associated with a worse prognosis in MF and SS; however, this thesis was not proven [69]. Folliculotropic mycosis fungoides is significantly more frequently identified in the head and neck area; thus, it is crucial to understand the prognosis associated with this MF subtype. Contrastingly to previous studies showing worse responses to therapy and worse course of the disease for patients with FMF, a substantial group with a good prognosis similar to early MF has been distinguished [4,134]. Clinically, the density of perifollicular infiltrate was shown to help in distinguishing indolent from aggressive FMF [4]. On the other hand, being aged over 60, large cell transformation and secondary bacterial infections were significantly correlated with a worse prognosis in this subset of patients [4]. The prognostic value of scalp involvement in other primary CTCLs is currently unknown.

Specific primary sites, such as the scalp, were found to have an unfavorable impact on the overall survival of patients with PCMZL [6]. On the other hand, PCFCLs have been previously shown to have a great prognosis with 5-year survival exceeding 95% [1,123,130,131]. Moreover, dissemination to extracutaneous sites is rarely observed, even when the lymphoma is not treated [49]. Most cases have an indolent course [1,123,130,131]. Interestingly, the PCDLBCL predilection site is not only the lower limb, but also the scalp and neck region [1]. The prognosis in sites other than the lower limb seems to be better for patients [132,133].

### 3.4. Clinical Presentation of CBCLs and CTCLs—An Overview

Scalp involvement in the course of PCLs may present a wide clinical presentation and mimic other neoplastic and inflammatory conditions. Knowledge of these presentations is crucial for clinicians. PCLs on the scalp may manifest as erythema, plaques, papules, nodules, and ulcerated/non-ulcerated tumors, which may or not be associated with alopecia [1,2,4,5,6,7,8,9,10,11,12,13,14,15,16,17,18,19,20,21,22,23,24,25,26,27,28,29,30,31,32,33,34,35,36,37,38,39,40,41,42,43,44,45,46,47,48,49,50,51,52,53,54,55,56,57,58,59,60,61,62,63,64,65,66,67,68,69,70,71,72,73,74,75,76,77,78,79,80,81,82,83,84,85,86,87,88,89,90,91,92,93,94,95,96,97,98,99,100,101,102,103,104,105,106,107,108,109,110,111,112,113,114,115,116,117,118,119,120,121,122,123,124,125,126,127,128,129,130,131,132,133,134,135,136,137,138,139,140,141,142,143,144,145,146,147,148,149,150,151,152,153,154,155,156,157,158,159,160,161,162,163,164]. Alopecia occurred in 69.0% of the analyzed patients.

Scalp evaluation is important in every PCL patient with skin lesions localized in different anatomical regions; however, it may be the only disease location. In the analyzed studies, plaque manifestation was the most common one, followed by patches, papules, and nodules [1,2,4,5,6,7,8,9,10,11,12,13,14,15,16,17,18,19,20,21,22,23,24,25,26,27,28,29,30,31,32,33,34,35,36,37,38,39,40,41,42,43,44,45,46,47,48,49,50,51,52,53,54,55,56,57,58,59,60,61,62,63,64,65,66,67,68,69,70,71,72,73,74,75,76,77,78,79,80,81,82,83,84,85,86,87,88,89,90,91,92,93,94,95,96,97,98,99,100,101,102,103,104,105,106,107,108,109,110,111,112,113,114,115,116,117,118,119,120,121,122,123,124,125,126,127,128,129,130,131,132,133,134,135,136,137,138,139,140,141,142,143,144,145,146,147,148,149,150,151,152,153,154,155,156,157,158,159,160,161,162,163,164]. In the course of PCL, both cicatricial and non-cicatricial alopecia may occur, in diffuse or patchy patterns [1,2,4,5,6,7,8,9,10,11,12,13,14,15,16,17,18,19,20,21,22,23,24,25,26,27,28,29,30,31,32,33,34,35,36,37,38,39,40,41,42,43,44,45,46,47,48,49,50,51,52,53,54,55,56,57,58,59,60,61,62,63,64,65,66,67,68,69,70,71,72,73,74,75,76,77,78,79,80,81,82,83,84,85,86,87,88,89,90,91,92,93,94,95,96,97,98,99,100,101,102,103,104,105,106,107,108,109,110,111,112,113,114,115,116,117,118,119,120,121,122,123,124,125,126,127,128,129,130,131,132,133,134,135,136,137,138,139,140,141,142,143,144,145,146,147,148,149,150,151,152,153,154,155,156,157,158,159,160,161,162,163,164]. Non-cicatricial, patchy alopecia has been noted more often [1,2,4,5,6,7,8,9,10,11,12,13,14,15,16,17,18,19,20,21,22,23,24,25,26,27,28,29,30,31,32,33,34,35,36,37,38,39,40,41,42,43,44,45,46,47,48,49,50,51,52,53,54,55,56,57,58,59,60,61,62,63,64,65,66,67,68,69,70,71,72,73,74,75,76,77,78,79,80,81,82,83,84,85,86,87,88,89,90,91,92,93,94,95,96,97,98,99,100,101,102,103,104,105,106,107,108,109,110,111,112,113,114,115,116,117,118,119,120,121,122,123,124,125,126,127,128,129,130,131,132,133,134,135,136,137,138,139,140,141,142,143,144,145,146,147,148,149,150,151,152,153,154,155,156,157,158,159,160,161,162,163,164]. Interestingly, alopecia was slightly more common in the CBCL group [1,2,4,5,6,7,8,9,10,11,12,13,14,15,16,17,18,19,20,21,22,23,24,25,26,27,28,29,30,31,32,33,34,35,36,37,38,39,40,41,42,43,44,45,46,47,48,49,50,51,52,53,54,55,56,57,58,59,60,61,62,63,64,65,66,67,68,69,70,71,72,73,74,75,76,77,78,79,80,81,82,83,84,85,86,87,88,89,90,91,92,93,94,95,96,97,98,99,100,101,102,103,104,105,106,107,108,109,110,111,112,113,114,115,116,117,118,119,120,121,122,123,124,125,126,127,128,129,130,131,132,133,134,135,136,137,138,139,140,141,142,143,144,145,146,147,148,149,150,151,152,153,154,155,156,157,158,159,160,161,162,163,164]. Furthermore, in FMF and SS groups, it was more frequent when compared to other types of CTCLs [1,2,4,5,6,7,8,9,10,11,12,13,14,15,16,17,18,19,20,21,22,23,24,25,26,27,28,29,30,31,32,33,34,35,36,37,38,39,40,41,42,43,44,45,46,47,48,49,50,51,52,53,54,55,56,57,58,59,60,61,62,63,64,65,66,67,68,69,70,71,72,73,74,75,76,77,78,79,80,81,82,83,84,85,86,87,88,89,90,91,92,93,94,95,96,97,98,99,100,101,102,103,104,105,106,107,108,109,110,111,112,113,114,115,116,117,118,119,120,121,122,123,124,125,126,127,128,129,130,131,132,133,134,135,136,137,138,139,140,141,142,143,144,145,146,147,148,149,150,151,152,153,154,155,156,157,158,159,160,161,162,163,164]. In the CBCL group, PCDLBCL was the variant in which hair loss occurred most commonly [1,2,4,5,6,7,8,9,10,11,12,13,14,15,16,17,18,19,20,21,22,23,24,25,26,27,28,29,30,31,32,33,34,35,36,37,38,39,40,41,42,43,44,45,46,47,48,49,50,51,52,53,54,55,56,57,58,59,60,61,62,63,64,65,66,67,68,69,70,71,72,73,74,75,76,77,78,79,80,81,82,83,84,85,86,87,88,89,90,91,92,93,94,95,96,97,98,99,100,101,102,103,104,105,106,107,108,109,110,111,112,113,114,115,116,117,118,119,120,121,122,123,124,125,126,127,128,129,130,131,132,133,134,135,136,137,138,139,140,141,142,143,144,145,146,147,148,149,150,151,152,153,154,155,156,157,158,159,160,161,162,163,164]. Clinical manifestations of scalp involvement in patients with PCL are shown in Table 3.

Cicatricial alopecia has been observed with a similar frequency, while other manifestations of alopecia (non-cicatricial alopecia in both patched and diffuse patterns) have occurred more commonly in the CTCL group. Furthermore, only erythema was observed with greater frequency in the CBCL group, while other manifestations were more frequently described in CTCLs, which is illustrated in Table 4.

#### 3.4.1. Clinical Presentation of PCLs Most Commonly Affecting the Scalp

##### Primary Cutaneous Follicle Center Lymphoma (PCFCL)

Primary cutaneous follicle center lymphoma is the most commonly reported CBCL localizing on the scalp. Clinically, it presents as erythematous papules, plaques, and/or tumors [25]. In rare cases, it may present as scarring alopecia, macular, or miliary agminated papules, or extensive telangiectasia of the scalp [25,68]. In one of the previous studies, non-scarring alopecic patches as a manifestation of PCFCL were found in 9% of cases [25]. Our analysis has shown that the mentioned percentage may be more substantial, as in 78.7% of cases included in this study, a sign of hair loss was found. Primary cutaneous follicle center lymphoma has also been shown previously to rarely be disseminated to extracutaneous sites [49].

##### Primary Cutaneous Diffuse Large B-Cell Lymphoma (PCDLBCL)

Primary cutaneous diffuse large B-cell lymphoma is the second most common CBCL found in the scalp area. In contrast to PCFCL, it presents more often with tumors, commonly with ulceration, and, less frequently, as papules/plaques [1,32,42,52,60]. Rarely, PCFCL may transform into PCDLBC, as reported in two patients with scalp involvement analyzed in the current study [28,49]. In both cases, PCFCL has been diagnosed using histology and immunohistochemistry and a stable course of the disease has been observed afterwards [28,49]. Subsequently, a progression of the disease clinically manifesting as rapidly growing tumors occurred [28,49].

##### Scalp Involvement in the Course of Other Cutaneous B-Cell Lymphomas (CBCLs)

Cutaneous B-cell lymphomas of other types rarely occur on the scalp. The most common variant is PCMZL, which has a predilection to appear on the trunk, upper extremity, and face [1,98]. PCMZL most commonly presents as small, reddish-purple, with single or multiple papules or nodules [6,26,98,153]. An interesting variant of CBCLs is B-lymphoblastic lymphoma (B-LBL), which is extremely rare; however, we have identified several cases, especially in patients below 18 years old, which appeared on the scalp [32,66,77,78,89,97,102,105,118,121,126]. B-LBL lesions are typically observed as red to purple nodules, which may subsequently develop into tumors within a few months [32,66,77,78,89,97,102,105,118,121,126].

##### Primary Cutaneous T-Cell Lymphoma of the Scalp

Primary CTCLs localizing on the scalp are a heterogenous group consisting of FMF, MF, SS, CD30+ lymphoproliferative disorders—pcALCL and LyP and other less common types. The majority of patients in the analyzed studies were FMF and classic MF; therefore, a detailed clinical presentation of these conditions is discussed below.

##### Folliculotropic Mycosis Fungoides (FMF)

Folliculotropic mycosis fungoides (pilotropic MF/folliculocentric MF) is the most common variant of MF, accounting for 10% of cases [2,135]. Clinical images may be of various and distinct clinicopathologic spectrums, which usually makes it a diagnostic challenge. The most important features are facial involvement, erythematous papules and plaques with follicular prominence, comedones, acneiform, and/or cystic morphology of the lesions [134,135,136]. Moreover, alopecia is also a frequent finding, often in the form of scarring [69,111,134,137,138]. In the advanced stages, the plaques are frequently observed with hair loss [139]. Sometimes FMF may also present as alopecia mucinosa, with areas of hair loss clinically resembling alopecia areata [5]. Accordingly, pruritus may be more frequent in more advanced stages of FMF [139]. In contrast to classic MF, the predilection to the head and neck is evident [4,134]. Interestingly, despite affecting this region of the skin, the lymphoma is rarely localized solely on the scalp [134].

##### Mycosis Fungoides (MF) and Sézary Syndrome (SS)

Our analysis shows that lymphoma-associated hair loss may be an important issue when considering that a majority of patients with MF and SS (66.7% of patients in the 142 reviewed studies) had at least a sign of alopecia. In contrast, in the study analyzing data from the American registry of 1550 patients diagnosed with MF/SS, only 38 (2.5%) had alopecia related to a lymphoma [69]. Two main clinical presentations have been identified—in the minority of the analyzed patients (34%), lesions presented as patchy alopecia, clinically resembling alopecia areata, while, in other patients, hair loss occurred within the lymphoma lesions [69,138]. In rare cases of erythrodermic MF and SS, total body hair loss has been observed [69]. According to previous observations, in these cases, hair may regrow after treatment implementation [46,55,92,108,112].

##### Scalp Involvement in Other PCLs

Other types of PCLs of the scalp are rare and we have identified only several cases involving the scalp in this literature review [15,19,20,31,33,36,39,40,43,45,48,50,65,71,73,75,85,86,88,96,109,110,113,120,125]. Of note, the frequently reported clinical presentation was a fast-growing reddish-brown ulcerated nodule [15,19,31,36,40,73,85,86,88,143].

**Figure 1:**
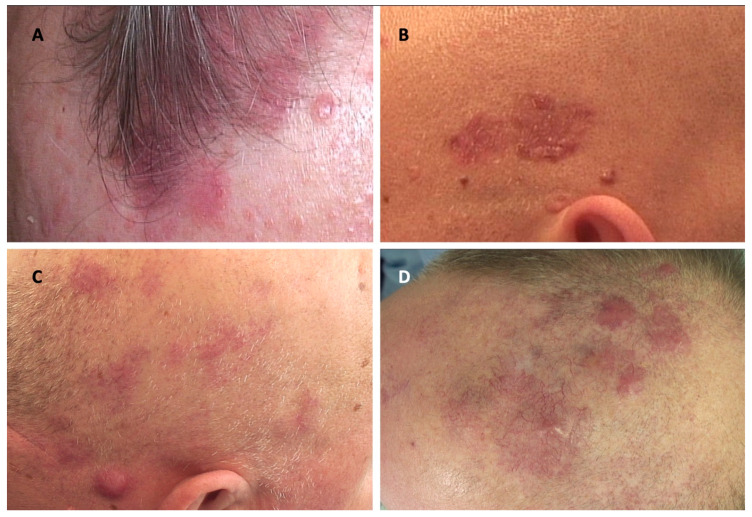
Clinical manifestations of cutaneous lymphomas affecting the scalp and their clinical mimickers. (**A**) Lymphomatoid papulosis; (**B**) multiple basal cell carcinomas; (**C**) primary cutaneous marginal zone lymphoma; (**D**) infiltration of the skin in the course of chronic lymphocytic leukemia.

**Figure 2:**
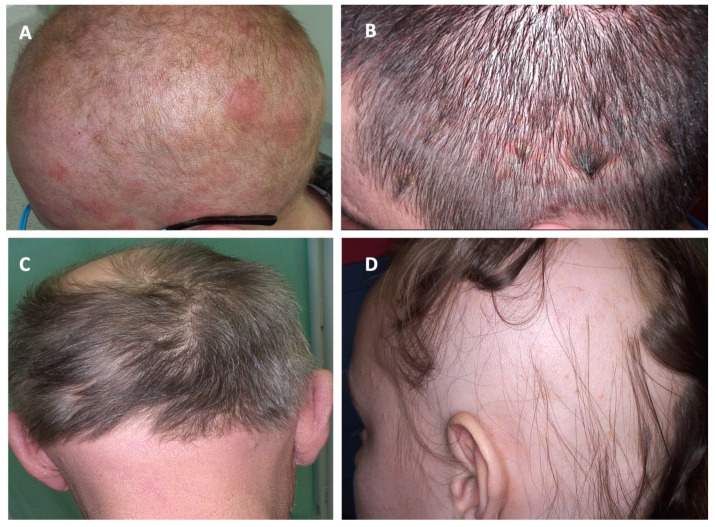
Clinical manifestations of cutaneous lymphomas affecting the scalp and their clinical mimickers. (**A**) Folliculotropic mycosis fungoides; (**B**) psoriasis; (**C**) folliculotropic mycosis fungoides; (**D**) alopecia areata.

**Figure 3:**
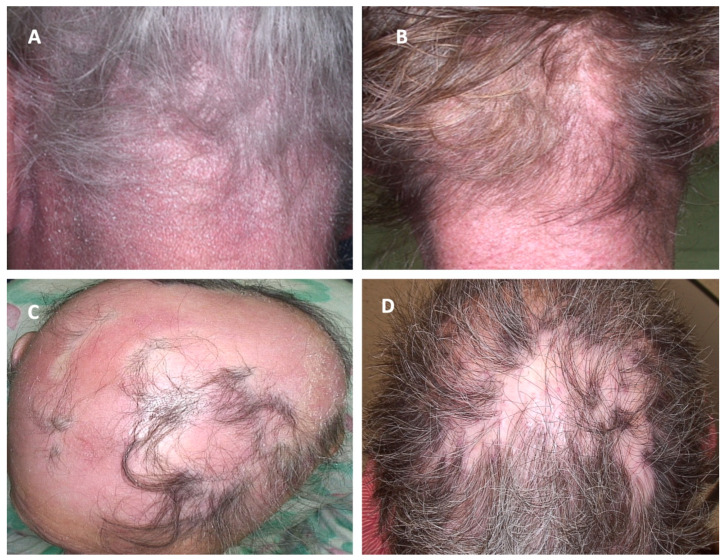
Clinical manifestations of cutaneous lymphomas affecting the scalp and their clinical mimickers. (**A**) Sézary Syndrome; (**B**) Dermatomyositis; (**C**) erythrodermic mycosis fungoides; (**D**) Discoid lupus erythematosus.

Various clinical presentations of PCLs of the scalp and other dermatoses affecting the scalp are presented in Figure 1, Figure 2 and Figure 3.

## 4. Diagnosis

### 4.1. Medical History

Despite extensive research on patients’ medical history, we have been able to find only a few that seem to be crucial in the pathogenesis of PCLs. In the PCLs of the scalp, only skin infections (especially of *Staphylococcus aureus* etiology) and the perpetual stimulation of lymphocytes by the bacterial antigens seem to negatively impact the prognosis [144]. On the other hand, chronic immunosuppression seems to be much more important in the development of B-cell non-Hodgkin lymphomas than in those of T-cell origin [119]. The lack of known risk factors directed medical history towards PCLs is difficult. In the case of PCMZCL suspicion, Borrelia burgdorferi infection has to be excluded [6]. During the process of obtaining a medical history, it is crucial to inquire about hair loss and previous therapeutic interventions, as a significant proportion of patients are initially diagnosed with an inflammatory dermatosis of the scalp. The influence of scalp involvement on the quality of life is important and should not be neglected. Importantly, women newly diagnosed with MF/SS and those with alopecia have been identified as having a particularly poor quality of life [163]. Patients with advanced disease or involvement of the head/neck, acral, or groin/genital sites also experience a significant impact on quality of life [165]. Therefore, patients with decreased quality of life should be supported with psychological counseling or camouflage (wig).

### 4.2. Physical Examinations and Laboratory Tests

In the early stages of PCLs extracutaneous signs are very rare. Most often “B” symptoms (night sweats, fever, and unintentional weight loss) are not noticed until the advanced stage of lymphoma [24,28,31,37,41,58,63,70,76,80,92,96,101,102,107,111,112,117,143]. Lymphadenopathy may be present and it is essential to carefully examine the lymph nodes of the palpable areas [2,13,20,21,24,28,30,33,34,37,42,43,48,49,52,53,57,58,62,63,66,70,71,72,73,74,76,77,84,86,89,92,94,97,99,102,103,104,109,114,116,118,119,135,138,143,145,146,147]. Laboratory test results are also within normal limits in the early stages of the disease [6,19,24,28,30,31,47,52,62,63,66,73,76,81,88,89,90,91,94,106,108,113,115,136,141,148,149]. In the advanced stages of the disease, the serum level of lactate dehydrogenase (LDH) may be elevated [14,24,28,32,43,48,51,52,61,62,70,72,89,90,102,115]. Previously, LDH concentration has been shown to be an important prognostic factor associated with increased mortality in advanced stages of MF [150].

### 4.3. Scalp Examination

Scalp assessment should be an integrative part of clinical examination in a patient suspected of or previously diagnosed with PCLs. The knowledge of a wide clinical spectrum is essential, and possible clinical manifestations have been described in the previous part of this review [149]. After assessment of the whole scalp area under good light conditions, additional examination with a dermoscope/videodermoscope should be performed, as it may reveal the details not visible to the naked eye.

### 4.4. Significance of Trichoscopy (Dermoscopy of the Scalp) in Diagnostics of PCLs

**Figure 4:**
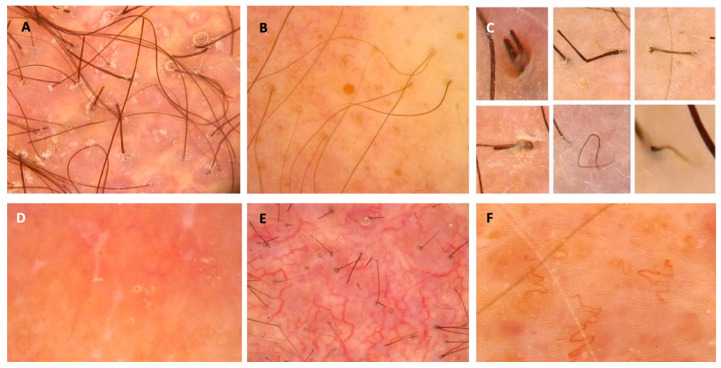
Dermoscopic features that may suggest the diagnosis of scalp lymphoma and should be an indication for biopsy. (**A**) Milky-red globules; (**B**) comedo openings; (**C**) different hair-shaft anomalies; (**D**) white dots and lines, salmon-colored background; (**E**) arborizing vessels; (**F**) tortous vessels.

The existing literature on dermoscopic manifestations of PCLs localized on the scalp is limited; however, some features have been described and may serve as diagnostic clues [10,12,14,22,24,50,55,148,153,154,160]. Dermoscopic features of PCLs have been recently summarized in a systematic review and, therefore, were not analyzed in our study [166]. In the mentioned article, the most commonly observed structures in classical MF were fine short linear vessels/linear vessels, spermatozoa-like vessels, and orange-yellow patchy areas [166]. In FMF, the most frequently observed lesions were comedonal lesions/comedo openings/central keratotic plugs and white halos around hair follicles/perifollicular accentuation [166]. The most common presentation of PCZML and PCFCL was a salmon-colored background with fine short/linear irregular/serpentine vessels [166]. Interestingly, in a multivariate analysis, orange structureless areas emerged as the strongest predictor of PCLs dermoscopy when compared with tumors and non-infiltrative inflammatory dermatoses [167]. This finding has not been reported in the reviewed articles describing dermoscopic manifestations of scalp PCLs apart from one study describing PCFCL in middle-aged females [10,12,14,22,24,50,55,148,153,154,160]. The most prevalent trichoscopic characteristics of erythrodermic CTCLs were the presence of numerous pili torti, numerous broken hairs, white thick interfollicular bands, and patchy hyperpigmentation of the background [12]. Dermoscopic features that may suggest the diagnosis of scalp lymphoma are presented in Figure 4.

## 5. Differential Diagnosis

Due to the wide clinical presentation of PCLs involving the scalp, the diagnosis may be challenging. It is important to examine the scalp of every patient with PCLs and perform further diagnostics in case of any suspicious findings. The integration of clinical assessment with trichoscopy helps to decide whether to biopsy. In addition, trichoscopy-guided biopsy seems to be helpful in the diagnostic process.

On the other hand, observation of new-onset alopecia that does not have typical features of other known alopecia subtypes will require total body examination and biopsy to exclude PCLs. The spectrum of inflammatory, infectious, malignant, and genetic disorders that should be considered in the differential diagnosis of scalp PCLs have been provided in Table 5 [5,69,168,169,170].

## 6. Treatment

The general treatment recommendations align with official recommendations and are therefore beyond the scope of this review. The recommendations do not include any annotations regarding any different treatment methods of PCLs localized on scalp skin [171,172,173]. Consequently, this review will focus on specific aspects that should be considered when seeking the optimal outcome for patients with PCLs localized on the scalp. It should be noted that some of the treatments employed in this location may result in permanent hair loss (surgery and radiotherapy).

## 7. Limitations

A publication and reporting bias in the literature cannot be excluded despite including all studies that analyzed PCLs of the scalp. Some of these studies lacked crucial clinical data and could not be included in the presented tables. Next, misclassification of the lymphoma due to changes in terminology and/or lack of pathologic pictures may have appeared, especially in older studies. The protocol of this study was not registered/made public ahead of the literature review.

## 8. Conclusions

The PCLs localized on the scalp encompass a broad range of potential clinical manifestations. In most cases, alopecia coexists with a diagnosis of PCLs on the scalp. Given the potential for poor prognosis with some of the more aggressive PCLs that can be found in this anatomic area, it is of paramount importance to emphasize a thorough physical examination. Early detection is critical to provide patients with the best chance for a favorable outcome.

## Figures and Tables

**Table 1:** Baseline demographic and clinical characteristics of the PCL patients with scalp involvement.

Characteristic	Number
**Total number of patients—no.**	**1482**
**Patients of known gender—no.**	502
Female—no. (%)	167 (33.2)
Male—no. (%)	335 (66.8)
**Mean age at disease onset [years]**	**49.1**
**Patients with cutaneous B-cell lymphoma (CBCL) no. (% of total)**	**1096 (73.9)**
Primary cutaneous follicle center lymphoma (PCFCL)—no. (% of CBCL no.)	711 (64.8)
Primary cutaneous diffuse large B-cell lymphoma (PCDLBCL)—no. (% of CBCL no.)	245 (22.3)
Primary cutaneous marginal zone lymphoma (PCMZL)—no. (% of CBCL no.)	107 (9.7)
Other CBCL—no. (% of CBCL no.)	33 (3.2)
**Patients with cutaneous T-cell lymphoma (CTCL) no. (% of total)**	**384 (23.8)**
Mycosis fungoides (MF)—no. (% of CTCL no.)	347 (90.4)
**Sézary** Syndrome (SS)—no. (% of CTCL no.)	10 (2.6)
Primary cutaneous anaplastic large cell lymphoma (PCALCL)—no. (% of CTCL no.)	9 (2.3)
Lymphomatoid papulosis (LyP)—no. (% of CTCL no.)	2 (0.5)
Other CTCL—no. (% of CTCL no.)	16 (4.2)
**Alopecia status during follow-up—no. of known cases**	**426**
Alopecia—no. (%)	294 (69.0)
No signs of alopecia—no. (%)	132 (31.0)

**Table 2:** Comparison of CBCLs and CTCLs with scalp involvement. ^a^—other CBCL subtypes have been classified as non-primary cutaneous marginal zone lymphoma (PCMZL), primary cutaneous follicle center lymphoma (PCFCL), primary cutaneous diffuse large B-cell lymphoma (PCDLBCL) and other CTCL subtypes have been classified as non-mycosis fungoides (MF), Sézary Syndrome (SS), Lymphomatoid papulosis (LyP), primary cutaneous large cell lymphoma (pcALCL).

Characteristic/Variable	CBCLs	CTCLs
**Total number of patients no. (%)**	**1096 (73.9)**	**384 (25.9)**
**Patients of known gender no.**	**123**	**379**
Female—no. (%)/Male—no. (%)	47 (38.2)/76 (61.8)	120 (31.7)/259 (68.3)
Female/Male ratio	0.6	0.5
Mean age at disease onset [years]	41.4	54.5
**Alopecia status during follow-up no./total no. of known cases (%)**	54/76 (71.1)	240/350 (68.6)
Alopecia in variants—variant no./total no. (%)	PCFCL, 37/47 (78.7)	MF excluding FMF, 32/48 (66.7)
	PCDLBCL, 9/10 (90.0)	FMF, 185/274 (67.5)
	PCMZL, 1/3 (33.4)	SS, 7/7 (100)
	Other CBCL ^a^, 7/16 (43.8)	PCALCL, 5/8 (62.5)
	-	LyP, 0/1 (0)
	-	Other CTCL ^a^, 11/12 (91.7)

**Table 3:** Clinical manifestations of scalp involvement in patients with PCLs. ^a^ Percentages of cicatricial versus non-cicatricial alopecia and patched versus non-patched alopecia. ^b^ Percentages of clinical manifestations of PCLs in cases of known status.

Characteristic	Number
Cicatricial alopecia no. (%) ^a^	40 (40.0)
Non-cicatricial alopecia no. (%) ^a^	60 (60.0)
Patched alopecia no. (%) ^a^	101 (87.8)
Diffuse alopecia no. (%) ^a^	14 (12.2)
Erythema no. (%) ^b^	53 (11.5)
Patches no. (%) ^b^	127 (27.7)
Plaques no. (%) ^b^	157 (34.2)
Papules no. (%) ^b^	124 (27.0)
Nodules no. (%) ^b^	109 (23.7)
Ulcerated tumors no. (%) ^b^	81 (17.6)
Non-ulcerated tumors no. (%) ^b^	82 (17.9)

**Table 4:** Comparison of clinical manifestations of CBCLs and CTCLs with scalp involvement.

Characteristic	CBCLs	CTCLs
Cicatricial alopecia no. (%)	20 (50.0)	20 (50.0)
Non-cicatricial alopecia no. (%)	17 (28.4)	43 (71.6)
Patched alopecia no. (%)	45 (44.6)	56 (55.4)
Diffuse alopecia no. (%)	2 (14.3)	12 (85.7)
Erythema no. (%)	34 (64.2)	19 (35.8)
Patches no. (%)	8 (6.3)	119 (93.7)
Plaques no. (%)	17 (10.8)	140 (89.2)
Papules no. (%)	23 (18.5)	101 (81.5)
Nodules no. (%)	29 (26.6)	80 (73.4)
Ulcerated tumors no. (%)	11 (13.6)	70 (86.4)
Non-ulcerated tumors no. (%)	17 (20.7)	65 (79.3)

**Table 5:** Differential diagnosis of scalp PCLs according to predominant clinical features.

Clinical Characteristic of Scalp PCL	Differential Diagnosis
Cicatricial alopecia	Frontal fibrosing alopecia Graham-Little syndrome Localized scleroderma (‘‘en coup de sabre’’) Discoid lupus erythematosus Cutaneous tuberculosis Cutaneous metastases Pseudopelade of Brocq Dissecting cellulitis Folliculitis decalvans Central centrifugal cicatricial alopecia Alopecia mucinosa Keratosis pilaris atrophicans faciei Post traumatic alopecia Primary skin cancer
Non-cicatricial alopecia	Alopecia areata Seborrheic dermatitis Atopic dermatitis Systemic and neonatal lupus erythematosus Trichotillomania Congenital erythrodermas associated with alopecia (Omenn syndrome, Netherton syndrome, severe combined immunodeficiency disorder)
Patchy alopecia	Alopecia areata Tinea capitis Graham-Little syndrome Localized scleroderma (‘‘en coup de sabre’’) Discoid lupus erythematosus Cutaneous metastases Neonatal lupus erythematosus Pseudopelade of Brocq Dissecting cellulitis Folliculitis decalvans Central centrifugal cicatricial alopecia Alopecia mucinosa Leprosy (Hansen disease) Secondary syphilis Trichotillomania Keratosis pilaris atrophicans faciei Radiotherapy Exogenous agents (selenium, MT-45)
Diffuse alopecia	Seborrheic dermatitis Atopic dermatitis Hypothoridism Trichotillomania Exogenous agents (chemotherapy, thallium, hypervitaminosis A, selenium, boric acid, thallium, arsenic, colchicine, mercury, MT-45) Systemic lupus erythematosus Favus (tinea favosa) Secondary syphilis Congenital erythrodermas (Omenn syndrome, Netherton syndrome, severe combined immunodeficiency disorder) Ectodermal dysplasia
Diffuse desquamative lesions	Seborrheic dermatitis Psoriasis Tinea capitis Pityriasis amiantacea Pityriasis rubra pilaris Secondary syphilis Congenital erythrodermas (Omenn syndrome, Netherton syndrome, severe combined immunodeficiency disorder) Acquired erythrodermas (psoriasis, eczema, drug eruptions, pityriasis rubra pilaris) Contact dermatitis
Erythema/macules and patches	Lichen planopilaris Psoriasis Graham-Little syndrome Discoid lupus erythematosus Neonatal lupus erythematosus Cutaneous tuberculosis Dissecting cellulitis Alopecia mucinosa Secondary syphilis Keratosis pilaris atrophicans faciei
Plaques, papules, nodules, and tumors	Cutaneous tuberculosis Leprosy (Hansen disease) Dissecting cellulitis Skin cancer Brooke-Spiegler Syndrome Naevus sebaceous of Jadassohn Cutaneous neoplasm metastases Skin involvement in the course of hematological malignancies

## Data Availability

The data presented in this manuscript are available in the PubMed database and on the sites of the cited article publishers.
